# Stakeholder-engaged process for refining the design of a clinical trial in home hospice

**DOI:** 10.1186/s12874-021-01275-0

**Published:** 2021-04-30

**Authors:** Jennifer Tjia, Margaret Clayton, Germán Chiriboga, Brooke Staples, Geraldine Puerto, Lynley Rappaport, Susan DeSanto-Madeya

**Affiliations:** 1 University of Massachusetts Medical School, 368 Plantation Street, AS6-2065, Worcester, MA 01605 USA; 2 University of Utah College of Nursing, Salt Lake City, UT USA; 3 University of Rhode Island School of Nursing, Kingston, RI USA

**Keywords:** Stakeholder, Home hospice, Clinical trial, Outcome measurement, Deprescribing

## Abstract

**Background:**

Clinical trials in home hospice settings are important to build the evidence base for practice, but balancing the burden and benefit of clinical trial conduct for clinicians, patients, and family caregivers is challenging. A stakeholder-engaged process can help inform and refine key aspects of home hospice clinical trials. The aim of this study was to describe a stakeholder-engaged process to refine, design, and implement aspects of an educational intervention trial in home hospice, including recommendations for refining intervention content and delivery, recruitment and enrollment strategies, and content and frequency of outcome measurement.

**Methods:**

A panel of interprofessional (1 hospice administrator, 3 nurses, 2 physicians, 2 pharmacists) and 2 former family caregiver stakeholders was systematically selected and invited to participate based on expertise, representing 2 geographically distinct hospices who were participating in the clinical trial. Teleconferences followed a predetermined procedural sequence: 1. pre-meeting materials distribution and review; 2. pre-meeting email solicitation of concerns in response to materials; 3. teleconference with structured and guided discussion; and 4. documentation and distribution of minutes for accuracy review and future meeting guidance. Discussion topics were distinct for each panel meeting. Written reflections on the stakeholder engagement process were collected from panel members to further refine our process.

**Results:**

Five initial biweekly teleconferences resulted in recommendations for recruitment strategy, enrollment process, measurement frequency, patient inclusion, and primary care physician notification of the patient’s trial involvement. The panel continues to participate in quarterly teleconferences to review progress and unexpected questions and concerns. Panelist reflections reveal personal and professional benefit from participation.

**Conclusions:**

An interprofessional stakeholder process is feasible and invaluable for developing home hospice intervention studies, contributing to better science, successful trial implementation, and relevant, valid outcomes.

**Trial registration:**

Clinicaltrials.gov, NCT03972163, Registered June 3, 2019.

## Background

Stakeholder engagement has emerged as an important component of meaningful clinical research [1–3]. Multi-stakeholder engagement, where diverse groups of stakeholders take part in the research project, is defined as “an iterative process of actively soliciting the knowledge, experience, judgment and values of individuals selected to represent a broad range of direct interest in a particular issue, for the dual purposes of creating a shared understanding and making relevant, transparent and effective decisions.” [4] While growing literature describes the stakeholder engagement process [1, 2, 5–7], methods and guidance for its use in palliative care and hospice research are limited [8–10], particularly in the important area of home hospice.

Clinical trials in home hospice are critical to building the evidence base for practice. However, balancing the burden and benefit of clinical trial conduct for clinicians, patients, and family caregivers is an ongoing challenge. This challenge is even more complex when designing a feasible study to be executed in home hospice that engages hospice patients and their family caregivers and presents an ideal situation for meaningful stakeholder engagement. Questions to be addressed include: the optimal design of a high-yield, low burden, clinical workflow-friendly approach to family caregiver-patient recruitment at hospice admission; selection and frequency of appropriate family caregivers and patient outcome measures; and heterogeneity of hospice team interactions that affect research implementation across different hospice sites.

To address these issues, a highly effective stakeholder engagement process was designed and implemented for an NIH-funded pilot randomized clinical trial, Standardized Patient Centered Medication Review (SPECTORx) in Home Hospice. The goal of SPECTORx is to reduce complexity and increase appropriateness of medication use for patients and family caregivers during home hospice care. In this paper, SPECTORx will be used as an exemplar to describe and share a stakeholder engagement method and approach in the refinement of clinical trial in home hospice. The Participatory Action Research (PAR) framework [11, 12], served as a foundation for the stakeholder engagement process. PAR is a research paradigm that puts stakeholders at the heart of the investigation process as collaborators who work together with investigators to resolve challenging issues. A key goal in PAR is authentic, bi-directional, and insightful engagement of stakeholders to enhance and enrich programmatic outcomes, in this case, the conduct of clinical trials to ensure study results are meaningful and clinically relevant to target populations. It is a collaborative, cyclical, reflective design process that focuses on problem solving, improving work practices, and understanding the effect of the research endeavor on the participating partners. The work of PAR typically includes cycles of planning, action, reflection, and evaluation/observation, with each cycle informing subsequent efforts. The intent of this manuscript is to describe our methodological approach to stakeholder engagement and to present the reflections of stakeholders regarding the value of this model. The implication is that our approach can be replicated and/or adapted by other investigators in the field of palliative care and hospice trial research.

## Methods

### Initial stakeholder engagement in SPECTORx - eligibility, recruitment and study setting

The goal was to assemble a key stakeholder panel, inclusive of interprofessional clinicians and family caregivers who participate in the care of home hospice patients. A multi-stakeholder panel consisting of 1 hospice administrator, 3 nurses, 2 physicians, 2 pharmacists, and 2 former family caregivers was systematically recruited and assembled to guide refinement of the study protocol and implementation by sharing their expertise and experience with hospice medication management. The composition of the panel was designed to include clinical, administrative, and front-line care delivery and care recipient perspectives. Deliberately recruiting a minority of physicians, equal in number to pharmacists and family caregivers, and fewer than nurses, was intended to mitigate the inherent power differential that can arise in interprofessional activities that include physicians [13]. The intention of having more than one family caregiver was to increase the weight of the family’s voice in the deliberation process relative to the clinicians’ voice. We did not include hospice patients on the panel because of their inherently vulnerable health status and limited life expectancy, which was much shorter than the planned time horizon of the stakeholder process. The final size of the stakeholder panel (*n* = 10) was also designed to be larger than the size of the investigator team (*n* = 6, 3 investigators plus 3 research staff) in order to avoid a negative imbalance in the ratio of stakeholder panelists relative to the investigator team during the meetings. To address geographic variation in clinical practice, we sought to include stakeholder panel members, in roughly equal number, from both of the 2 geographically distinct hospice agencies participating in the SPECTORx pilot trial in the US.

Individuals were eligible for the stakeholder panel using the following criteria: hospice administrators (i.e. executive or clinical director) actively employed by the hospice agency; nurses employed by the hospice (i.e. not per-diem) and providing > 24 h/week of in-home clinical care; physicians serving as the hospice medical director; pharmacists providing services primarily to the hospice; and former family caregivers needed to be English-speaking, have provided > 50% of care for a family member who died > 3 months from the time of recruitment and be without complicated grief by the Inventory of Complicated Grief scale [14].

### Structured meeting procedures

The interprofessional and family caregiver stakeholder panel was convened every 2 weeks for 3 months prior to the start of the pilot trial using a structured procedure for teleconferences. The panel met with the entire investigative team during each meeting. Meetings were designed to follow a cyclic structure that fostered a sense of familiarity within the panel and allowed each member to better understand the panel process of soliciting input from all stakeholders. For example, each meeting opened with a welcome and greeting, followed by a very brief summary of decisions to date, then an introduction of the meeting topic followed by points for discussion related to the day’s topic. After panel discussions about design and implementation procedures were completed in the first 4 bi-weekly meetings, the research team proposed, and the stakeholder panel agreed, to continue meeting quarterly to review unexpected questions and concerns about the content of the SPECTORx intervention, informational materials, and trial protocol.

**Fig. 1:**
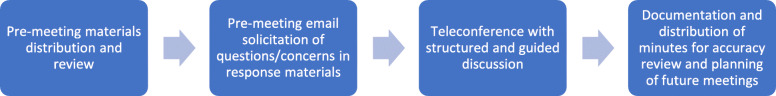
Process for Stakeholder Meeting Material Review and Documentation

The initial team meeting established relationships and negotiated rules for engagement. To facilitate relationship building, the initial meeting started with personal introductions which included stories about why the panelists were involved with the project based on personal experiences. Subsequent meetings started with personal check-ins, and then followed a sequence of procedures for appropriate and efficient review of key program questions. This sequence is listed in Fig. 1. The video teleconference meetings were digitally recorded for accuracy of minutes. To acknowledge the time commitment and opportunity cost that each meeting entailed, all participants were sent a $50 electronic gift card following their participation in each 1–1.5-h stakeholder engagement session. This amount was selected to be equal across all participants and not be coercive, with the intent of communicating value to each member.

### Meeting topics

The goal presented to the stakeholder panel was to finalize a pre-screened trial protocol prior to conducting the SPECTORx medication intervention trial [15]. Stakeholder recommendations were solicited in a sequential fashion to: a. refine content and delivery of the SPECTORx intervention, b. ensure recruitment and enrollment strategy was acceptable and feasible, and c. determine content and frequency of outcome measurement. Stakeholder panel members were presented with general information about each topic and key questions to guide the discussions described herein (Table 1).

**Table 1:** Key Content of SPECTORx Hospice Educational Intervention

1. Family Caregivers Support for Medication Management	
2. Determining Medication Appropriateness in Hospice	
3. How to Have Deprescribing Conversations	

### SPECTORx content and delivery

While the key content of the intervention focused on 3 topics selected a priori*,* (Table 2) Panel discussions were used to gain insight into educational content delivery, including issues regarding provision of CE/CEU/CME, targeting of frontline hospice clinicians, and how to best deliver the education (e.g. in person vs online). Panel questions included: What do you already do well? What is challenging now? What would be helpful to emphasize? The development of ancillary educational materials, such as patient/family caregiver pamphlets and clinician pocket reference cards, and how to implement an online community of practice for hospice clinicians was also discussed.

**Table 2:** Participatory Action Research (PAR) Informed Approach to Stakeholder Panel Engagement, by Clinical Trial Stage

PAR Cycle Phase	Stakeholder Panel Start-Up	Trial Stage		
		Refining Protocol and Design	Trial Execution	Dissemination
**Plan**	• Recruit key members • Plan timing and process/ procedures	• Define a priori protocol and design questions for panel input	• Plan for updates and check-in	• Plan for interpretation of analysis/results
**Action**	• Define rules for engagement and communication • Personalize approach to facilitate inclusion and participation	• Present goals, questions/challenges • Invite additions to agenda setting from panel • Execute recommendations from prior cycles PAR cycles (if applicable)	• Present updates for recruitment, acceptability, feasibility	• Present interim analysis/results • Engage in plan for presentation and dissemination
**Reflection**	• Discuss, reflect and make recommendations for refining process	• Discuss, reflect and make recommendations for challenging issues, addressing gaps	• Invite feedback on successes and challenges	• Invite interpretation of interim analysis and results
**Evaluation/ Observation**	• Record process and minutes from stakeholder meetings • Check in with panelists and investigator team	• Record process and minutes from stakeholder meetings • Review of protocol by investigators, IRB, study safety officers, and stakeholders	• Record process and minutes from stakeholder meetings • Measures of trial benchmarks (e.g. retention, adherence to intervention)	• Record process and minutes from stakeholder meetings • Peer review of research manuscripts and meeting presentations

### Recruitment and enrollment strategies

The optimal design of a recruitment strategy that is high-yield, low burden, and clinical workflow-friendly for family caregiver-patient dyads in home hospice admission is unclear. Recruitment and attrition are inherently challenging issues in palliative care research [16, 17] While family caregiver-patient dyads have been successfully enrolled in prior home hospice studies [18, 19], this typically occurred at the 4th or later home visit [11]. Further, while a two-step recruitment process that starts with a brief, standardized, ‘interest in research’ screen in the ‘hospice admission packet’ to refer family caregiver-patient dyads for recruitment by trained research staff [20], widespread acceptability is not established.

Some evidence suggests that universal screening on admission is an effective procedure [20]. Our goal was to refine the protocol so that it allowed for enrollment of family caregiver-patient dyads as close to hospice admission as possible, before many medication changes occur. Panel questions included: When and where recruitment should start; who should conduct recruitment; what level of involvement from hospice clinicians is acceptable; how to minimize burden on hospice staff; what past experiences for recruiting have worked well at your site; what is the correct order for referral for recruitment; and should enrollment be by telephone or an in-home visit?

### Selection and frequency of family caregiver outcome measurement

Weighing the burden and benefit of administering different family caregiver outcomes assessments is critical as family caregivers are already burdened with the grief of their loved ones’ illness and their caregiving responsibilities. Options discussed with the panel included primary data collection using well-validated family caregiver measures [21], and the use of secondary data such as the Consumer Assessment of Healthcare Providers and Systems (CAHPS®) [22], a hospice assessment that is routinely collected to measure consumer satisfaction. Key candidate family caregiver measures for stakeholder panel consideration were the family caregiver Medication Administration Hassle Scale [23] and the Patient-Centered Care Communication Survey [24, 25]. Panel questions included: Should we measure outcomes among patients? If so, how often should we measure patient outcomes?

### Selection and frequency of patient outcome measures

Selection of appropriate patient outcome measures was also challenging due to our intent to enroll a heterogeneous population whose hospice admitting diagnoses have different trajectories [26] and varied abilities to self-report. Unpredictability of disease trajectories [26, 27] affect measurement frequency decisions and levels of missing data. Different abilities to self-report affect selection of disease-specific versus generic quality of life (QOL) instrument choice [28]. A key question was whether to *only* use medical record data for patient outcomes since these can be obtained with no burden to the patient. Panel questions included: Should we measure outcomes among patients? If so, how often should we measure patient outcomes?

### Soliciting reflections of panelists regarding the stakeholder engagement process

After the design process was completed and the trial was underway, we asked the stakeholders to reflect on their participation and consider authoring a manuscript describing their experience. While all of the stakeholders were interested, some found this to be intimidating. To ease their concerns and facilitate the paper-writing process, the research team provided panelists with several written prompts about their involvement, including, for example: How engaged were you in the process? What are the benefits of this panel to nurses, staff, patients, and caregivers? What challenges were there in participating in the stakeholder panel or in the stakeholder process? Comprehensive synthesis of those reflections is beyond the scope of this paper, but a few responses are included herein to provide empiric data about the stakeholder experience, including successes and challenges.

## Results

Five stakeholder meetings were held prior to the start of the pilot trial. The first meeting encompassed introductions and an overview of the clinical trial and study aims. The second meeting focused on SPECTORx intervention content and delivery, including measurement burden and frequency. The third meeting focused on recruitment, informed consent, and data collection. The fourth meeting focused on the proposed content and delivery of family caregiver materials. The fifth meeting presented the refined content and protocol incorporating stakeholder panel feedback. The meetings lasted 60–90 min and all but one meeting was attended by an average of 9 stakeholder panel members.

### Stakeholder engagement in the refinement of the clinical trial protocol

#### Recruitment and enrollment strategy

Stakeholders were helpful in recruitment protocol refinement, providing a sounding board for variations in procedures that needed to be developed to respectfully accommodated differences between the study sites. Flexibility and tailoring the recruitment and enrollment procedures for participating hospice organizations and even individual sites was important for the success of this trial as each hospice had its own workflow and procedures for patient admission and enrollment. Further, the two hospices differed in their philosophical approaches to medication management and deprescribing, with one making changes very close to admission, while the other waited to make changes over the ensuing weeks. While all members of the stakeholder panel provided valuable insight into recruitment and enrollment strategies, the clinician stakeholders, most notably the nurses and physicians, had recommendations based on their prior interactions and experiences with patients and family caregivers in practice and research. Their recommendations were further validated by the family caregiver stakeholders. Decisions regarding telephone vs in-person recruitment and enrollment were also informed by differences between the two hospice organizations. Specifically, in-person recruitment was very challenging in one hospice organization serving a rural community. There was universal agreement for developing recruitment procedures that did not rely on front-line nursing staff to minimize nurse burden.

When presented with the option of universal screening on admission [20], the idea was initially rejected by our stakeholder panel as they felt patients and families needed time to adjust to home hospice care and that approaching them with an invitation to participate in a research study up front could initially be overwhelming. However, at the time of trial initiation, one hospice had adopted a version of universal opt-out for patients and families using an informational letter and opt-out form in all admission packets. This allowed a research assistant to conduct recruiting calls among patients and families who did not opt-out. In summary, our stakeholder panel agreed that local flexibility was the key recommendation to support successful recruitment and enrollment.

#### Selection and frequency of outcome measurement

##### Family caregiver

All members of the stakeholder panel felt that assessing family caregiver medication difficulties was a key outcome measure for the study. They recommended a frequency of every 2 weeks in the first month, and then every 4 weeks. In addition, panel members recommended assessing satisfaction with hospice care following study completion (i.e. post-death of the family member).

##### Patient

Stakeholder panel members suggested evaluating medication use and changes for patients, as well as health service utilization (e.g. emergency department visits and hospitalizations). All members of the stakeholder panel clearly stated that direct patient engagement for data collection was critical in this trial because the patient is the most important element of the entire project. They felt strongly that quality of life and symptom burden also be collected if the patient was willing, and weighed in on the frequency of measurement (i.e. every 2 weeks in the first month, and then every 4 weeks until study completion or patient death). Input from former family caregivers was especially helpful during these panel discussions.

#### Stakeholder input for the educational intervention

##### Content

A detailed description of the content of the nurse driven SPECTORx educational intervention and patient-family caregiver materials is beyond the scope of this paper. Stakeholder input provided insight into augmenting key educational content in the following ways: 1. it was very important to reinforce communication about deprescribing and to use established “off the shelf” communication training materials, such as those from End-of-Life Nursing Education Consortium (ELNEC); and 2. staff training must address developing patient/family caregiver trust, including challenges in home hospice admission and how to build it. Stakeholder panel members also felt it was important to cover communication regarding messaging of the trial so that staff felt comfortable with recruitment and participation. Finally, panel members suggested that pharmacists would benefit from additional information including rationale for the deprescribing recommendations and tapering/discontinuation protocols.

Another unanticipated recommendation from the stakeholders was the creation of patient/family caregiver facing materials with medication-related information. This was led by a subcommittee from the stakeholder panel and resulted in a key additional intervention product for the clinical trial. This subcommittee was facilitated by a co-investigator and included a nurse, pharmacist and family caregiver, to address concerns from both the clinical and patient/family perspective.

##### Delivery

For feasibility of delivery in the busy lives of front-line clinicians, nurse stakeholders, with agreement from the other clinician stakeholders, recommended that the duration of hospice staff training for the SPECTORx intervention should be short, with educational delivery for no longer than 20-min per session. For example, a 1-h training module was divided into 3 sessions of 20-min duration. It was also recommended to have asynchronous online delivery to maximize use and ease of completion.

#### Reinforcing content and retention

Stakeholders suggested that content could be accessed and reinforced through electronic access via smartphones for content, in addition to summaries of the educational material on paper.

### Stakeholder panelists’ reflections on the stakeholder process

In individually solicited written responses from the panelists, members shared that they felt engaged in the stakeholder-engaged research design and process in a few ways: personally, through collaboration and teamwork, and in identifying the purpose and tasks to come out of the meetings. This sentiment was common: “I feel I was very engaged in the process.” Reported benefits to nurses, staff, and caregivers included being involved in producing a usable tool for deprescribing education for hospice staff and family caregivers, being able to share and receive a diversity of viewpoints, and ultimately helping patients and family caregivers in reducing medication burden. The most notable challenges with the process were finding and coordinating time to participate in stakeholder meetings, and juggling competing with external clinical demands. Interestingly, at least one stakeholder felt there were no challenges with the process. An unexpected outcome of the process was that many stakeholders shared that they gained some personal and professional skills as a result of our meetings. In unsolicited feedback, individual stakeholders expressed appreciation for being asked to be part of the stakeholder panel and having their input respected, valued, and incorporated into the clinical trial protocol, and for family caregivers, being able to process and use their experience to help others receiving home hospice.

## Discussion

In this paper, we illustrate a PAR-grounded approach to stakeholder engagement using a federally-funded pilot clinical trial, SPECTORx, as an exemplar. The success of our process, exemplified by the generation of practical recommendations that strengthened the study’s trial protocol, outcome measurement, and intervention content, as well as the relationship-building that fostered enthusiastic buy-in for executing the trial and spawned new collaborative projects, prompted reflection on the process to identify key elements to consider using in other palliative care and hospice trials going forward. We summarize these stakeholder engagement elements in the context of the typical PAR cycle with stages of Planning, Action, Reflection and Evaluation. (Table 2) While the cycle stages are presented linearly, it is important to note that the elements are cyclical and can evolve organically to reflect the developing processes of the collaborative work. For example, iterative rounds of action and reflection may occur before a stage of evaluation.

In the stakeholder planning and start-up phase, we note that intentional recruitment of key players is essential to success. These include stakeholders who have interest and unique knowledge of a topic and have power to make a difference. For meaningful engagement, we invited the stakeholders into the process of defining the structure and rules for communication, as well as take a personalized approach to ensure their inclusion and participation. We invited stakeholders and investigators to reflect on tentative meeting procedures before execution, and then recorded the recommendations for agreement and final review. This helped to foster a sense of shared commitment to the values and objectives of the stakeholder engagement process. We accomplished these tasks prior to and during our first stakeholder meeting, before any discussion of research content issues.

Throughout our process, the research team was sensitive to the historical imbalances of academic-participant collaborations [29], hierarchical imbalances arising from combining healthcare professionals and lay persons to the same team, and interprofessional power imbalances deeply ingrained in healthcare systems [13]. Within the UK context our design process might be labelled as ‘co-production’ of research [30, 31], with egalitarian participation and voice being the goal throughout the research design process from beginning (i.e. the research question) to end, we describe our process as collaborative [30], with the role of the stakeholders being two-fold: adding valuable knowledge and skills to the research process, and providing experiential information about the hospice environment in which the study would be implemented and research findings will be used [32]. As such, while our communication process was meant to be as egalitarian as possible, the stakeholders served in a consulting role providing contextual expertise, and the research team drove the research process, without “threatening the legitimacy of the scientific endeavor” [30].

.For example, in the pre-trial stage of refining the protocol and design of the trial, we defined the a priori questions for the panel in the planning stage, and then presented them and invited additional agenda items from the panel. The built-in flexibility to possible gaps in the investigator team’s scope of perceived trial challenges highlights a key benefit of inviting others to the clinical trial planning process. We engaged in a number of cycles of ‘action, reflection and evaluation’ so that each of the main areas of review received adequate focused discussion and actionable recommendations.

We are continuing with stakeholder engagement into the trial execution phase, by presenting the final protocol and intervention content, and having periodic meetings to present updates about key trial benchmarks such as recruitment and acceptability of the intervention. With a goal of transparency, we presented both success and challenges with the intent to reflect back the outcome of the prior PAR process and to solicit input on refinements to the path forward.

We plan to, and recommend that others, continue the stakeholder engagement process through the dissemination stage. We include interpretation of analysis and interim results in this stage and recommend inclusion of the stakeholder panel in the planning of the scientific and non-scientific presentations both internal to the participating study sites and external to scientific consumer. In each stage, we continue to use a structured and efficient system for documenting and collating the input from the stakeholders to reinforce transparency, authenticity, and integrity to the process.

Most of the elements offered above are not inherently novel, and are presented and organized differently in other models of stakeholder engagement [1, 2, 5, 6]. However, we would argue that this cyclical 4-stage model to be practical, intuitive, and adaptable to the various types of studies including clinical trials [12]. We found that our process resulted in clinical practice-grounded protocol refinement and an enhanced educational intervention for our trial.

An examination of 50 PCORI trials requiring stakeholder engagement noted challenges to an engaged stakeholder process [6]. Most notable was the lack of time for meetings. Language difficulties when levels of expertise and experience varied were also noted. Using this knowledge, the research team spent considerable time in building a structured, yet flexible, method for stakeholder engagement; thus challenges reported in other studies were not as present in our process. This can be attributed to our intentional efforts from the onset to foster a sense of participation and community in panel discussions despite our geographic distance. These efforts included demonstrating respect for all opinions, soliciting opinions of those who were more reticent, scheduling regular meetings in advance, using a predictable meeting structure, emailing an agenda and discussion materials prior to the meeting to allow participant preparation, and distribution of minutes following the meetings to ensure all viewpoints were accurately captured.

We note several limitations to our stakeholder engagement process. First is that our process was limited to 2 home hospice agencies in the US. Mitigating this limitation is that they were geographically diverse and represented both urban and rural populations. Second is that we did not include patient participants in this panel because we were concerned that the burden of engagement and the time horizon for the project did not fit well with extremely limited life expectancies. In lieu of this, we recruited former family hospice caregivers who could reflect and represent the patient point of view. Third is that the trial focused on medication management. However, we believe this process can work for other types of trials as there is nothing inherent about medications that should influence the stakeholder engagement process.

In conclusion, we demonstrate that meaningful, interprofessional stakeholder engaged process is feasible and invaluable for home hospice intervention studies. Our deliberate approach to selection of well-represented stakeholder panel of interprofessional team members and family caregivers, and predetermined procedural meeting structure, is a method for optimally engaging key hospice stakeholders to refine and successfully implement a clinical trial in home hospice with an overarching goal of ultimately improving palliative and end of life research approaches. We believe the implication for palliative care research is that it can contribute to better science, justification for measures, and ultimately better clinical outcomes. The process can also make for more effective intervention content and relevance to clinical partners, patients and family caregivers. Together scientists and stakeholders can work together to develop relevant and appropriate research to build an evidence base for practice that will optimize end of life care for patients and their caregivers.

## Data Availability

Data sharing is not applicable to this article as no datasets were generated or analyzed during the current study.

## Abbreviations

SPECTORx: Standardized Patient Center Medication Review
PAR: Participatory Action Research
CE: Continuing Education
CEU: Continuing Education Unit
CME: Continuing Medical Education
PCORI: Patient-Centered Outcomes Research Institute
